# The American Health Resort Association

**Published:** 1892-08

**Authors:** J. D. Hartly

**Affiliations:** Corresponding Sec’y; 1204 Milwaukee Ave., Chicago, Ill.


### THE AMERICAN HEALTH RESORT ASSOCIATION.

*Journal of the American Medical Association*, July 16, 1892.

This Association met at the Tremont House, Chicago, June 30, and held three sessions.

There were present delegates representing Canada, Michigan, Massachusetts, Wisconsin, Florida, New Hampshire, New York, Pennsylvania, California, Illinois, Vermont, Colarado, Texas, Iowa, New Mexico and Central America.

A large correspondence was read by W. A. Chatterton, Secretary, from the absent members in various parts of the country.

The President, T. C. Duncan, M. D., of Chicago, then delivered a lengthy address, in which he outlined the good work of the Association, and how it was appreciated by the profession, enabling them to select climates adapted for the various cases of consumption. From reports received from the winter points, New Mexico had proven the most satisfactory. This is of interest to the profession who are trying to save some of the "hundred thousand consumptives" who die annually in this country.

Dr. J. F. Danter, of Toronto, Canada, read a paper on the "Climates and Resorts of British America."

A report on the climate of Manitoba was read, from Dr. Clark, of Winnipeg.

The climate of New Brunswick was presented by Dr. J. Z. Currie.

From these reports it seems that there are a large number of consumptives in Canada, especially in the Eastern provinces.

Dr. Adam Miller read a paper on "Sun-spots and magnetic influence in Disease."

The climate of Nebraska was presented by Dr. Brown.

The climate of California and its resorts was presented in papers by Drs. J. D. Hartley and S. W. Andrews, of Chicago.

Dr. W. P. Roberts, of Boston, read a report on the climate of New England, in which he reported that 15,000 die annually there from consumption.

"Consumption in Michigan" was the subject of a paper by Dr. Veenboer.

Dr. O. W. Gordon, of Council Bluffs, reported his disappointment in visiting various resorts, and spoke highly of New Mexico.

A report from Dr. A. Petin, of Las Cruces, N. M., formerly of Paris, was read, in which he said they had almost constant sunshine, less than two inches of precipitation in twenty-eight months, and that consumptives sent there were all doing well.

A report on the Adirondack region was read from Dr. Skinner.

Dr. B. W. James, of Philadelphia, contributed a paper on "Climate Maxims."

The climate of Costa Rica was presented by Dr. Buchanan.

Dr. A. S. Butler reported on the climate of Honduras.

"Texas as a Resort for Consumptives," was the title of a paper by Dr. Marshall.

Reports on mineral waters were presented from Las Vegas Hot Springs, N. M., Eureka Springs, Ojo Caliente Hot Springs, N. M., Costa Rica and Londonderry.

Prof. I. N. Danforth gave an address on "Mineral Waters, their Analysis and Uses."

He said the profession was being imposed upon by imperfect and fraudulent analysis. In the first stage of Bright's disease he thought that bland water should be used, and in the second lithia waters.

Prof. W. S. Haines made a valuable report on "Bacteria in Mineral and Potable Waters." In some mineral waters he found two bacteria to the cubic centimeter, and in some drinking water he found as high as 8,000.

A large number of members were admitted.

It was reported that a Congress of Climatologists would meet in Chicago next year, and it was voted that the Association meet with it.

The following officers were elected: T. C. Duncan, M. D., President, Chicago; J. F. Danter, M. D., first Vice-President, Toronto, Canada; I. N. Danforth, M. D., second Vice-President, Chicago; W. P. Roberts, M. D., third Vice-President, Boston, Mass.; T. S. Hoyne, M. D., Treasurer, Chicago; W. A. Chatterton, Recording Secretary, Chicago; J. D. Hartley, M. D., Corresponding Secretary, Chicago; W. W. Van Baun, M. D., Philadelphia; Prof. W. S Haines, M. D., Chicago.

The full proceedings and papers will be published shortly, and all members will be supplied with these valuable and interesting transactions. For further particulars, address

J. D. HARTLY, M. D., Corresponding Sec'y.

1204 Milwaukee Ave., Chicago, Ill.

